# Children Learn, Children Do! Results of the “Planning Health in School”, a Behavioural Change Programme

**DOI:** 10.3390/ijerph18189872

**Published:** 2021-09-19

**Authors:** Margarida Vieira, Graça S. Carvalho

**Affiliations:** Research Centre on Child Studies, University of Minho, 4710-057 Braga, Portugal; graca@ie.uminho.pt

**Keywords:** health promotion, health education, Transtheoretical model, eating behaviour, obesity prevention, children’s health

## Abstract

The ‘Planning Health in School’ programme (PHS-pro) is a behavioural change intervention to assess and improve the eating habits of children, particularly the intake of fruit and vegetables, and to guide them towards healthy choices. The programme and its educational components are based on the Transtheoretical Model of stages of change to integrate nutritional literacy and build up problem-solving and decision-making skills. Children (*n* = 240, ages 10–12) of one large suburban school in Porto’s metropolitan area (Portugal) were evaluated throughout PHS-pro implementation during one school year in a repeated time–series design. Children’s outcome evaluations were conducted through seven 3-day food records for nine eating behaviour, documented after each learning module and through participatory activities which analysed attitudes, preferences and expectations. Changes were observed in children’s eating behaviour, supported by changes in motivation as perceived in their attitudes and expectations. Significant changes were found in a higher consumption of vegetable soup (*p* = 0.003), milk products (*p* = 0.024), and fruit (*p* = 0.008), while the consumption of high-energy dense food (*p* = 0.048) and soft drinks (*p* = 0.042) significantly decreased. No positive effects on fried food, water, vegetables and bread consumption were found. The PHS-pro intervention proved to be effective in developing healthy eating behaviour in young people.

## 1. Introduction

A healthy diet is one of the key elements to ensure proper growth during childhood [1,2]. However, at present, it is abundantly evident that most children have unhealthy dietary habits, mainly because they do not eat fruit and vegetables (F&V) in sufficient quantities, while consuming excessively high-fat snacks and high-sugar foods and beverages [3,4,5,6]. 

The scientific literature warns of the negative impact of such eating patterns, particularly low F&V intakes, which are associated with obesity and chronic severe diseases, such as diabetes, cardiovascular disease and cancer [7,8,9]. Additionally, eating sweet candy and cake, as well as drinking soft drinks regularly, are identified as risk factors for obesity and early childhood caries, two related health problems affecting children worldwide [10].

A primary consideration is to encourage children to eat healthily to ensure the adequate dietary intake of central nutrients and micronutrients, based on a wide diversity of food sources, wherein F&V have a large share [11]. A healthy diet can provide all the conditions for optimum growth during childhood and adolescence and prevent all forms of poor nutrition to move away from the main risk factors of the obesity epidemic and related chronic diseases [12]. For this reason, helping children in the changing process for better eating behaviour is now a much-needed measure, and school-based health promotion programmes are clearly identified as the most effective strategy to promote healthy behaviour among young people [13,14]. 

Schools have been identified as the best settings to implement interventions to educate young people and promote healthy habits [15]. The strong argument for health interventions in schools is that children can be reached there with relatively less effort and ensured global coverage. There are, however, more advantages: (i) children spend the larger part of the day at school, and for about nine months per year, reflecting the importance of school in children’s lives; (ii) children usually eat at least one meal and two snacks in the five weekdays, which can influence the eating patterns and the way of living; (iii) the school allows for the combining education and health, and these two components can interact and be cooperative for learning and promoting health, but also have effective results such as health knowledge and skills improvement; and (iv) the school can provide the opportunity for children to be physically active and eat healthy foods [16,17,18].

A systematic review [19] assessing 55 international studies reporting health promotion interventions for preventing obesity in children found strong evidence to support beneficial effects on children’s health, particularly programmes targeted at children aged 6 to 12 years. Of the 55 examined studies, positive changes were found in children’s eating behaviour in 20 studies regarding nutrition knowledge, eating practices, F&V consumption, energy-dense snack foods, sweetened/carbonated drinks, sweet foods and other indicators of better diets [19]. More recently, another systematic review [20] restricted to randomised controlled trials (RCTs) included 153 RCTs of the programmes which aimed to prevent obesity in children aged from 0 to 18 years. Of the 153 RCTs, 45 programmes targeted children aged 6 to 12 years, showing some evidence that diet combined with physical activity interventions may be effective in obesity prevention and reinforcing that a healthy diet and a physically active lifestyle have many health benefits beyond the promotion of a healthy weight. 

Bearing this in mind, a school-based prevention obesity intervention called the ‘Planning Health in School’ programme (PHS-pro) was designed, implemented and evaluated on children attending school grade 6, aged 10 to 12 years old. The PHS-pro aimed to improve children’s eating behaviour and active living with the implementation of a nutritional education programme shaped in eight learning modules and delivered monthly over a full school year.

The PHS-pro provided easy-to-use food and nutrition knowledge for healthy choices, increasing skills for better decision-making and nutrition literacy, and was shown to be effective in improving children’s nutritional statuses by several anthropometric measures [21].

This study aims to determine the impact of the eight learning modules on children’s eating behaviour and lifestyle over a 1-year school intervention. Additionally, it provides detailed information about how children’s behaviour and attitudes improved and under which circumstances. The effectiveness of the programme was evaluated according to the selected key behaviour goals of the learning modules, and children’s eating behaviour changes were assessed using a 3-day food record following each learning module. 

## 2. Materials and Methods

### 2.1. Study Design

**Figure 1:**
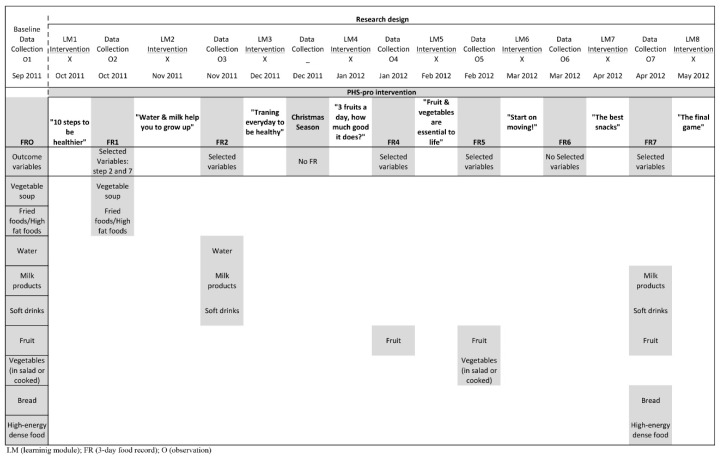
Research design and analysed variables according the learning modules’ goals.

In order to achieve the objectives of this longitudinal study, the research design was based on a repeated time–series design. The time–series strategy consisted of taking a series of initial observations and introducing a variable or a new dynamic into the research field, after which another series of observations were made and in which only one group was available [22,23]. Hence, a baseline data collection was conducted before the intervention, followed by a series of repeated measures to determine the influence of the independent variable (the PHS-pro) to provide evidence about its effects on children’s eating behaviour (see Figure 1). 

The study period extended from September 2011 to May 2012, and the data of the present study corresponded to the implementation of the programme content over one school year. The PHS-pro learning modules were implemented three weeks after the baseline data collection and evaluations were conducted at monthly intervals except in two distinct periods: in December, the Christmas month, to avoid the effects of the festive season which can cause changes in the usual diet; and in May, after the last learning module since the evaluation period overlapped with the last days of school, making it impossible to control the return of the usual data collection. 

The Scientific Council of the Institute of Education of the University of Minho approved this study, and ethical permission was obtained from the Pedagogical School Board. Furthermore, teachers’ class coordinators and the Health Promoter Office (HPO) of the intervention school made themselves available to offer assistance in developing the research activities. 

To initiate the PHS-pro, several arrangements were carried out to evaluate the school setting environment: the food infrastructures of the school with respect to the bar/buffet, vending machines, cafeteria and the food menus; nutrition and health-related school curriculum; physical education classes; and health prevention initiatives. Aside from the celebration of World Food Day, the school did not have any initiatives or awareness campaigns for healthy behaviour. Thus, a partnership was established with the HPO of the intervention school, enabling the inclusion of the PHS-pro in the Yearly Activity Plan of the school. In addition, the HPO coordinator conducted all the arrangements required for implementing this intervention in the school setting, connecting the PHS-pro with several stakeholders: class coordinator teachers, grade 6 teachers, school principals, the school parents’ association and the school canteen, in order to guarantee the availability of F&V at lunch with an extra salad buffet.

Parents were invited to participate in the longitudinal study and were fully informed of its content and objectives. All parents signed the informed consent form. A similar procedure was delivered to children in the classroom on the first day of school. Detailed information was first conveyed orally, then through an individual distribution of a paper package that included participants’ information sheets to explain the research purpose and the child’s informed consent to enable free and active participation, beyond the formal request previously addressed to parents.

### 2.2. Participants 

All grade 6 children (*n* = 240, ages 10–12) of one large suburban school in Porto’s metropolitan area (Portugal) participated in this study. Of these, 11 (4.6%) were excluded from the study: one refused to participate, two moved to another school and eight were special educational needs cases. Consequently, the sample size at the onset of this study was 229 children. However, at the end of the study, the sample was reduced to 157 children (with 87 girls and 70 boys) due to failure to meet the following criteria: (i) having complete baseline data evaluation (corresponding to the food record of zero); (ii) having returned two or more complete food records over the intervention; (iii) having parents’ informed consent and their informed consent for participation. 

### 2.3. Intervention

The implementation of the programme was designed upon the Transtheoretical Model (TTM) to encourage eating behaviour changes and engage children to participate actively in this process of change [24]. 

The TTM provides an appropriate framework to build up the educational content of PHS-pro, expecting that children would progress through the five stages identified by Prochaska and his collaborators [24,25]: (i) precontemplation—no intention to change behaviour or take action in the near future, unawareness of their problems; (ii) contemplation—aware of unhealthy habits, interest and intention to change in the next 6 months; (iii) preparation—intending to take action in the next 30 days; (iv) action—making changes and visible modifications on specific behaviour targets along 6 months; (v) maintenance—working to continue and consolidate healthy habits. In addition, the core constructs of TTM (processes of change, decision balance, self-efficacy) were applied for supporting progress change across stages and facilitate behaviour’ change over the PHS-pro [26,27]. Based on this, the intervention consisted of eight learning modules, which followed the five-stages of TTM readiness, for moving children from inaction to action or to maintenance, in order to a successful behaviour change. The process of behaviour change was assessed through activities developed over the learning modules, and the progress on dietary behaviour was monitored through the application of a 3-day food record after each module, which provided children’s feedback.

The intervention consisted of eight learning modules focused on the four main goals of the programme, which were based on the international guidelines of the WHO [28]: adequate consumption of five servings F&V/day; decreasing high-sugar food and beverage intake (to 10% of free sugar of total daily energy); decreasing high-fat and energy-dense food consumption; 1 h of physical activity and no more than 2 h spent watch TV.

The children chose eight topics about food, eating and active living out of a list of 16 to be used for the learning modules, following the participatory methodology principles [29,30]. Each learning module (LM) emphasized a different topic with a specific behaviour change, and was designed to last 45 min with one scheduled per month as part of the Natural Science classes over the academic year. The learning modules sequence was as follows: LM1 (“10 steps to be healthier”); LM2 (“Water & milk help you to grow up”); LM3 (“Training every day to be healthier”); LM4 (“3 fruits a day, how much good it does?”); LM5 (“F&V are essential to life”); LM6 (“Start on moving!”); LM7 (“The best snacks”); LM8 (“Final game: who has learned about everything?”—a programme overview). 

The learning modules goals were to provide nutritional education, identify obstacles and training problem-solving skills, to support children to find solutions and strategies, and motivate them to set a personal behaviour change goal and implement it in daily life. All the modules were conducted to follow the TTM stages of change and the processes of change. Additionally, in each LM, a participatory activity was included for supporting children to enhance skills and make decisions to encourage healthier behaviour. This kind of activity enabled children to participate, as well as to receive feedback about specific behaviour change goals. Table 1 shows the eight modules, describing the scope and the sequence of activities (column 1), the goals (column 2), the behavioural change goals and the expected outcomes in the food records (column 3).

### 2.4. Outcome Measures

To measure the eating behaviour of children over the implementation of the educational components and to evaluate behaviour changes after each LM and over the intervention, a 3-day food record was selected to assess all the food and beverages that children consumed over 3 days. The 3-day food record was considered the most accurate method, both in qualitative and quantitative terms, to describe the food consumed [31,32]. Nevertheless, measuring food intake among free-living individuals continued to be a challenging task [33].

Seven 3-day food records were applied to assess the intervention efficacy. The first collected data, for the baseline, were obtained before the intervention and were designated food record zero (FR0), followed by six more 3-day food records applied subsequently to each learning module (FR1, FR2, FR4, FR5, FR6 and FR7). In other words, after each learning module children had to record within the following days the respective 3-day food record. The exceptions for returning the food record were for the LM3, corresponding to the Christmas season, as there were always changes in eating habits during Christmas, with typical food consumptions which varied considerably compared to the usual pattern, and for the LM8 in the last days of the academic year. Instead, a small questionnaire was applied to children to identify any changes which occurred in their eating and lifestyle behaviour over the intervention. Likewise, the LM6 topic concerned sports practice and active living and was not a food topic. Accordingly, the food record (FR6) was applied, yet was also used as a possible follow-up evaluation.

The 3-day food record form was introduced to children in the classroom with detailed instructions on how to fill it out correctly and the delivered rules were defined. The instructions of the food record form provided representative pictures of household measures (cups, spoons or standard packaging) to elucidate the definitions of portion sizes and to serve as an example of the completed records. This helped children to record all foods and beverages consumed during three consecutive and subsequent days after each LM. We encouraged the children to record events at the time of eating and to use their best judgment in the reports. 

In the current study, only children who returned the completed FR0 (baseline data) plus at least two or more completed food records were reviewed and validated by the nutritionist and considered for analysis.

#### 2.4.1. Measuring Eating Behaviour Changes

The 3-day food records data were coded following the behavioural coding protocol [34]. Given that people consume foods, not nutrients, the PHS-pro had a behavioural focus instead of a knowledge-based focus. Therefore, to guide children towards desirable nutrition-oriented behaviour changes and healthy food choices, rather than nutrients [35], the first step was to categorise all food items reported by the children into a behavioural perspective to assess the eating behaviour changes outcomes. 

All food items reported in the seven 3-day food records were coded by meal and place of consumption, food category, cooking method, and the number of servings (one serving was the default serving and the usual amount of food recorded by children).

Each food item was categorised in terms of providing central nutrients (proteins, vitamins, minerals, dietary fibre, complex carbohydrates, fats, free sugars) based on the six food categories of the New Portuguese Food Guide [36]: (i) fruits; (ii) vegetables; (iii) potato, cereal, and cereal products; (iv) pulses, milk and dairy products; (v) meat, fish, seafood, and eggs; (vi) fats and oils. Subcategories were created within each of these groups, except for vegetables and fruits, in a total of 21 items. Food items were identified within each subcategory as low-, medium- and high-fat options or low-, medium- and high-sugar options. Two examples of these were: French fries and potato chips, which were credited as high–fat options, compared to boiled potatoes that were credited as low fat, and donuts or cakes, which were credited as high-sugar foods compared to fresh bread. Additionally, typical Portuguese mixed dishes had to provide at least one cup of cooked vegetables or legumes to be credited as containing one serving of vegetables, for example: ‘chicken and vegetable stew’.

After these procedures, to assess the servings of specific foods and beverages consumed, and to measure behaviour changes, smaller eating sub-behaviour categories were created according to the learning modules goals, and nine study variables were organized for analysis: 

(1) Vegetable soup servings: this included all traditional Portuguese preparations of vegetable soups. In LM1 (“10 steps to be healthier”, see Table 1), all children chose to take action and go up to step 2, which corresponded to an increase in their vegetable soup consumption at meal times and an increased intake of vegetables. The LM5 (“Fruits & vegetables are essential to life”, see Table 1) was also dedicated to the vegetable topic, in which vegetable soup was considered an important preparation for increasing vegetable intake. Consequently, this variable was assessed by comparing FR0 (baseline data) with FR1 and FR5.

(2) Fried food/High-fat food servings: this included all fried foods and products, such as French fries, chips, rissoles, pasties, croquettes, fish fingers, and nuggets. In LM1 all children chose to take action and go up to step 7, which corresponded to avoiding eating fried foods. This variable was assessed by comparing FR0 with FR1.

(3) Water servings: this included tap water and bottled water. Improving water consumption as the first choice of beverage was developed in LM2 (“Water & milk help you to grow up”, see Table 1). This variable was assessed by comparing FR0 with FR2.

(4) Milk products servings: this included plain milk, milk mixed with coffee or barley, and yoghurt, and excluded chocolate milk. LM2 was dedicated to the healthy and adequate consumption of milk products. Additionally, LM7 (“The best snacks”, see Table 1) focused on milk products as healthy choices for smaller snacks. This variable was assessed by comparing FR0 with FR2 and FR7.

(5) Soft drinks servings: this included soda, cola, iced tea, and added-sugar squash juices. In LM2 the soft drinks’ topic was developed to promote a decrease in children’s consumption of soft drinks. Again, the LM7 strengthened the same idea of avoiding and limiting these beverages on snacks. This variable was assessed by comparing FR0 with FR2 and FR7.

(6) Fruit servings: this included all fruits and fresh fruit juices, except fruit in syrup. LM4 was exclusively dedicated to the fruit topic (see Table 1). Then, it was further strengthened in LM5, together with vegetables, and in LM7 as one of the best choices for children’s snacks. Therefore, the fruit variable was assessed by comparing FR0 with FR4, FR5 and FR7.

(7) Vegetable servings: this included all vegetables, legumes, salads, greenery, and mixed dishes rich in legumes or vegetables, and excluded potatoes. LM5 was entirely dedicated to the vegetable topic. This variable was assessed by comparing FR0 with FR5.

(8) Bread servings: this included all varieties of fresh and toasted bread. LM7 explored the best nutritional options for children’s snacks, in which bread had a healthy place. This variable was assessed by comparing FR0 with FR7.

(9) High-energy dense food servings: this included croissants, panikes, donuts, cakes, pastries, cookies and biscuits. LM7 explored the best nutritional options for children’s snacks compared with other food choices loaded with fat and sugar. This variable was assessed by comparing FR0 with FR7.

The variables were limited to the behaviour of these nine foods, which were particularly relevant to the learning modules goals (see Table 1) and to the overall objectives of the PHS-pro. Figure 1 provides a summary of the analysed variables according to the learning modules goals.

#### 2.4.2. Measuring Attitudes, Preferences and Expectations

Participatory activities were implemented to support peer-led activities and children’s decision-making over the learning modules. Such activities were also used to collect data related to children’s attitudes, preferences and expectations. Different activities were developed for each of the eight learning modules to support children in changing their attitudes and behaviour towards the learning modules goals (see Table 1). 

The feedback returned by the children in the classroom was then subjected to content analysis.

The eight participatory activities are described below.

Activity one (A1): Choosing the step, changing for the better.

Children were asked which of the 10 wise steps presented in LM1 was the most difficult. As mentioned above, two steps were equally chosen: step 2, increasing vegetable soup servings on meals; and step 7, related to avoiding fried products. The children were also asked to elect the step that they found most prepared them for immediate behaviour change. Children’s commitments were based on the improvements of their daily behaviour for the same two steps, 2 and 7. The children’s responses were given orally and recorded on the feedback activity sheet. Children’s attitudes towards change behaviour were assessed through this activity, and feedback was evaluated in connection with the FR1. 

Activity two (A2): Favourite beverage of the month.

From the discussion about the pros and cons of beverages usually consumed by children, it was proposed that children chose the favourite beverage of the month, and the result of this was: adopting water or milk instead of soft drinks. The children’s responses were recorded on the feedback activity sheet. Children’s attitudes towards change behaviour were assessed throughout this activity, and feedback was evaluated in connection with the FR2. 

Activity three (A3): Planning for a healthier family.

Children were invited to analyse their families’ lifestyles and design what they considered to be of the highest importance for changing their home environment to develop a healthier family. A card was individually delivered to each child at the end of this activity, which asked them to return it after the Christmas holidays. This activity allowed analysing whether children could identify the risky eating behaviour of their own families and develop problem-solving skills. 

Activity four (A4): My fruit planning: take a step forward into the future.

Children were asked to identify and record the fruit servings that they consumed the day before, during the present day, which fruits they would like to eat the next day, or even whether they would like to taste a new fruit. Three cards were delivered to each child to registering their feedback. This activity allowed for the analysis of fruit servings consumed and assessed children’s expectations of fruit serving consumption for the next day. 

Activity five (A5): The most popular ones: soup, salad and side dishes.

Children were asked to report their food preferences regarding the choice between vegetable soups, salads or vegetable side dishes. During this activity, three cards were delivered to each child to note down their three favourite options. The preferences of vegetable options were assessed.

Activity six (A6): What sort of sports do you play?

Children were asked which sports or playful activities they usually practised outside school, in which environments they practised sport, and whether this was within a family context. Additionally, children were asked which activities they would most like to have the chance of trying. A card was individually delivered to collect children’s feedback regarding different sporting environments. The attitudes towards sports participation and playful activities were assessed, as well as children’s expectations of which sports they would like to try.

Activity seven (A7): Small meals, big impact.

Snack choices that children considered to be a healthy snack were assessed with a two-step approach: before implementing the LM7 (“The best snacks”, see Table 1) and immediately after it. To each child two cards were applied for the two moments. The attitudes towards change behaviour were assessed through this activity.

Activity eight (A8): The final game: who has learned about everything? 

Children were invited to play a little game called ‘let’s play on: who has learned about everything?’. The children’s responses were given orally and recorded on the feedback activity sheet by the researcher with the assistance of the teacher. This activity allowed children’s self-evaluation of their attitude and behaviour changes which occurred in their eating habits over the PHS-pro.

### 2.5. Statistical Analysis 

Descriptive statistics were calculated to describe participants’ characteristics at baseline using the mean and standard deviation for continuous variables and frequencies for categorical variables. 

In this study, only children who delivered baseline data (FR0), and at least two post-intervention food records (FR1, FR2, FR4, FR5, and/or FR7) were considered for analysis.

To assess the effects of the intervention of the outcome variables representing different food behaviour, an analysis of the Wilcoxon signed-rank test was performed to examine differences between pre-intervention (baseline: FR0) and post-interventions (FR1, FR2, FR4, FR5 or FR7). Results were expressed as the median and interquartile range (IQR).

Initial analyses were performed for the symmetrical distribution of the outcome variables by analysing the histogram. 

A *p*-value < 0.05 was accepted as statistically significant for all analyses, and the statistical software SPSS (package version 21) was used to perform them.

Children’s feedback of the activities, which developed during the learning modules, were treated as categorical variables and analysed in a descriptive way, and no statistical testing strategy was used. Furthermore, content analysis was applied to the qualitative responses of children to the open-ended questions [37]. 

## 3. Results

Of the 229 grade 6 children, 157 (68.6%) completed the baseline 3-day food record (FR0) and two or more subsequent food records. Therefore, only data for these 157 children were used in the analysis to minimise the unreliability of the dietary outcome measures. The participation rates in the data collection of the other six 3-day food records applied during the intervention were 91.1% in FR1, 92.4% in FR2, 87.9% in FR4, 82.2% in FR5, 59.2% in FR6, and 80.9% in FR7. Considering that the LM6 was dedicated to the sports topic and active living, and since the FR6 had a relatively low returning with the worst children’s participation rate, the FR6 data was withdrawn from the analysis. 

### 3.1. Baseline Characteristics of Participants 

The mean age of the children was 10.9 years (SD = 0.6), 55.4% of whom were female with no statistical differences detected between genders (χ2 = 1.84; *p* = 0.175).

Table 2 summarises the baseline eating behaviour of children according to the nine food groups. The most common significant dietary concern was the F&V consumption. Children reported an intake of F&V well below the recommended levels over a 3-day period. At baseline, more than one third of the children reported not eating fruit (31.2%) or vegetable soup (36.9%) and more than half did not eat any sort of vegetables (51.0%) over 3 days. Milk products had a regular intake, except for 1.9% of children who reported no consumption and 3.2% who consumed one portion over 3 days. Bread intake was also common for snacks but not for 4.5% of children who reported not eating it. High-energy dense food intake was not reported by 17.8% of children. However, more children ate at least one or two servings of these foods over the 3 days. Finally, about 39.5% of the sample reported eating fried foods once in 3 days. 

Daily water consumption was not a regular behaviour for 14.6% of children, who reported not drinking water over 3 days. However, soft drinks consumption was already established, as a current habit among young people, as one can see in more than half of children (51.0%, i.e., counting the frequencies of children who reported daily servings), reporting the intake of one or more servings over the 3 days that were analysed. 

### 3.2. Changes in Food Behaviour

Table 3 shows the changes in the behaviour of nine foods by comparing children’s food intakes between baseline (one month before the intervention) and the corresponding 3-day food record. Regarding the two goals of LM1: increasing vegetable soup and decreasing fried food servings, children reported in their FR1 a significant change in vegetable soup servings by doubling the median value from 1 to 2 servings/3-day period (*p* = 0.003) between baseline and the FR1. For the fried food variable, there was no significant change. 

Following the LM2 goals, an increase in water and milk consumption and a decrease in sugar-sweetened soft drinks in the FR2 was expected, but only milk consumption had a statistically significant change (*p* = 0.024). Though the median value was equal at baseline and FR2, higher milk consumption was reported in 75 (51.7%) out of the 145 children analysed in the FR2 compared to baseline, and in 25 children (17.2%), milk quantities were equally consumed (data not shown). Soft drinks did not show significant changes (*p* = 0.340) between the two moments, although there was a reduction in the first quartile (Q1) from 1 to 0 servings over 3 days, indicating that 25% of children decreased the intake of soft drinks. 

A significant increase in fruit consumption was observed (*p* = 0.008) following LM4. Indeed, more children ate fruit, indicated by a median value of 2 fruit servings and showing an increase in the third quartile. In fact, of the 138 children cases analysed, 67 (48.6%) reported a higher number of fruit servings at FR4 compared to baseline, and 35 children (25.4%) reported equal fruit servings. 

Vegetable soup, fruit and vegetable servings were examined between baseline and FR5, as increasing the consumption of these foods was the main goal of the LM5. There were no significant changes in these consumptions. However, vegetable soup preserved the positive tendency as, in the 129 cases analysed, 51 children (39.5%) reported eating more vegetable soup in the FR4 than in the baseline, and 38 (29.5%) retained an equal consumption. Likewise, children did not report changes in fruit consumption; nevertheless, the third quartile maintained the number of fruit servings observed in the previous evaluation at FR4. 

The vegetables topic was again the main focus of LM5, and increasing consumption was the most important goal, yet no positive changes were found in vegetable servings. On the contrary, vegetables (in a salad or cooked as a side dish) recorded relatively low consumption, where half of the children did not eat any kind of vegetables over a 3-day period at both baseline and FR5 evaluations, except for the vegetable soup that maintained the usual intake.

Regarding the five food variables evaluated following the LM7 (bread, high-energy dense food, milk products, soft drinks and fruit), consumption changes were found significantly different between baseline (FR = 0) and FR7 for high-energy dense food and soft drinks. After the intervention with LM7, children significantly decreased high-energy dense food from a median of 2 to 1 servings/3-day period (*p* = 0.048). For soft drinks, children reduced servings of this kind of beverage, with half of the children consuming no soft drinks in a 3-day period. Bread and milk products had no changes and maintained the usual consumption. Similarly, fruit consumption had no significant change, yet the consumption of fruit decreased among 58 children (45.7%) of the 127 children cases observed (data not shown). 

In short, regarding the nine studied variables, children improved their nutritional practices in five variables (vegetable soup, milk products, fruit, high energy-dense food and soft drinks) over the PHS-pro intervention. 

### 3.3. Changes in Attitudes, Preferences and Intentions towards Healthy Food Choices

The participatory activities were introduced during the learning modules implementation to promote both children’s in-group discussion and active participation. 

The first two activities (A1 and A2) allowed children to show their attitudes toward behaviour change. For example, in A1, children decided to improve two steps (instead of one as initially proposed) that were equally chosen: step 2, advocating the increase in vegetable soup intake; and step 7, advocating the avoidance of fried foods. This attitude was followed by a significant change in vegetable soup servings found in FR1, already referred to above. However, regarding step 7, there was no change, although children had initially indicated their positive attitude towards changing. 

In A2, children’s results showed an increase in the intake of water consumption and milk and a decrease in the intake of sugar-sweetened soft drinks. This attitude was followed by a significant change in servings of milk products observed in FR2, but not in water or soft drinks consumption.

In the third activity (A3), children were asked which key strategy would be an effective plan for promoting a healthy family. Of the 157 children participating, 118 (75.2%) returned the card, in which each child, individually, identified the risky eating behaviour that their own family should avoid. Four children (3.4%) reported parents needing to quit smoking as the primary strategy to promote a healthier family, 97 (82.2%) children reported healthy eating at home, and 12 (10.2%) expressed physical activity as the most important for a healthy family. Five children (4.2%) did not clearly specify a strategy and merely stated, “My family needs to change habits”. Clearly, children were able to determine the key changes for a healthier family environment, and most of them expressed the need to take specific measures to change eating habits at home.

In the fourth activity (A4), 142 children (90.4%) returned the feedback card of fruit servings consumed and their fruit eating intentions. Table 4 shows the results of the detailed analysis. On the day before this assessment, more than 35.9% of participants reported the intake of two fruits, and almost one quarter (23.9%) the intake of three fruits; 11 children (7.7%) still did not eat fruit. For the assessment day, more than one quarter (25.4%) of children recorded one serving fruit intake; nevertheless, the time of assessments ranged over the school day, between morning and afternoon, which meant that it could not be confirmed whether the same fruit intake would follow the profile of the previous day. 

Children’s intentions to eat fruit for the next day showed highly positive results, as nearly half of the children (49.3%) stated that they intended to eat three servings of fruit the next day. According to this, a great number of children (25.4%) showed the intention of improving fruit intake, and all children had a positive attitude to improve fruit consumption. 

In the fifth activity (A5), children reported preferences of vegetables (Table 5) with the 156 children (99.4%) providing feedback, from the total sample of 157. A proportion of 78.2% of children reported having two favourite vegetable soups, green cabbage soup and carrot soup, the two most indicated by children. For fresh salads, the majority (66.7%) had at least two preferences (lettuce and tomato salads, either separated or mixed); however, five children (3.2%) expressed no appreciation for any kind of salad. Vegetable servings as a side dish received a similar acceptance, with a large percentage of children (68.6%) reporting two preferences: broccolis and peas. In addition, more children indicated having three, four or five preferences for this kind of vegetable preparation compared with vegetable soups or salads. 

Activity six (A6) applied during the LM6 focused on active living such as physical and leisure activities. Table 6 summarises the 152 children’s reports (96.8%) from the total of 157 participants. 

The great majority of children were not engaged in sports practices (56.6%). When asked which activities they usually dedicated their time to in the playground, playing games was the most mentioned activity (51.3%). After school, 18 children (11.8%) indicated not completing any kind of physical activity, but the rest used the time after school to play different leisure activities. Regarding weekends with the family, more than one quarter of children (25.7%) reported not completing any kind of leisure activity or sports, while 44.1% indicated “going for a walk” as the most frequent family activity. Finally, when children were asked about activities they would like to have the chance of trying, the majority showed an interest in practising some sports (65.8%) or playful activities (31.6%); between 34 activities mentioned by children, surfing was the most popular (with 16 occurrences), followed by tennis (12), horse-riding (11), and skating (11). These preferences showed that the majority of children were motivated to engage with sports and other activities compared with the great percentage of children (56.6%) who reported not practising any kind of physical activity.

In the seventh activity (A7), children were assessed regarding foods that could be part of a healthy snack. Table 7 shows the differences between the two assessment moments of A7. Of 157 children, 155 delivered the two cards applied (98.7%), and among these, a great number of children correctly identified healthy foods for snacks, with 127 (81.9%) making snack choices such as bread and milk products instead of high-energy dense foods and soft drinks. In contrast, 28 children (18.1%) selected unhealthy choices for their snacks (high-energy dense foods, soft drinks, and fried foods). Nevertheless, of the 127 children that made healthy choices, 55 (35.5%) did not include fruit as a healthy snack on the first card assessment (A7-card 1). After the LM7, a change in children’s attitude was found in 148 children (95.5%) reported in all healthy foods, including fruit servings, against seven children (4.5%) who continued with the previous snack choices which did not include fruit. Accordingly, reported healthy snack choices improved after A7.

The last activity (A8) gathered children’s feedback about the concepts of food topics developed over the seven learning modules. Of the 157 children distributed by nine classes, four classes had difficulty recalling the concepts of the topic developed during the third learning module (LM3): training every day to be healthier. However, all the concepts of the other learning modules were 100% recorded, suggesting an increase in the healthy-eating knowledge with the step-by-step learning modules approach.

## 4. Discussion

This study aimed to determine the impact of eight learning modules on children’s eating behaviour regarding nine foods (fruit, vegetable soup, vegetables in a salad or cooked, milk products, bread, high-energy dense food, fried food, water and soft drinks) and their responses to one learning module dedicated to active living over the PHS-pro intervention.

Results showed substantial consumption changes with a statistically significant increase in vegetable soup, milk products and fruit. At the same time, high-energy dense food and soft drinks significantly decreased between the interval of evaluation (from baseline to post-intervention learning modules). In contrast, fried food, vegetables, water and bread consumption had no significant change. 

These findings indicated that PHS-pro implemented over the school year successfully met some of the selected eating behaviour goals. However, it cannot be expected that, if continued over time, the programme could positively influence the other children’s eating behaviour, which might help prevent and reduce the development of obesity.

The most disappointing results of the study were the lack of significant changes in fried food and vegetable servings (in salads or cooked). This can be explained by the fact that these foods are usually served at meals not prepared by children, and such factors either facilitate or halt such behaviour since children do not have absolute control in such circumstances [38]. In contrast, fruit serving consumption significantly increased after the PHS-pro intervention. Reinaerts [38] explained that fruit consumption is more under children’s control, and if the child chooses to eat fruit, availability is the only important factor for fruit consumption. 

As mentioned above, at baseline, children recorded a relatively low vegetable consumption, whereas half of the children did not eat any kind of vegetables over a 3-day period. This is a matter of serious concern regarding children’s health. Given that adolescence is an accelerated growth period and vegetables are the most major sources of vitamins, minerals and dietary fibre, an adequate intake of vegetables containing these nutrients, which are crucial for healthy growth, should be encouraged to sustain healthy habits that are of greater long-term significance for preventing obesity and other chronic diseases. Several studies provided evidence in support of these important mechanisms [11,39,40]. Therefore, these results make the intervention programmes for promoting vegetables appear even more important, and their continuity must be ensured as long as children are growing because these programmes might be the only effective strategy to face this perplexing trend [13].

In general, the results found in this study support the findings of similar studies for children with the same age range, as reviewed by Water et al. [19]. High levels of F&V intake were found in five intervention studies. Reductions in high energy-dense foods were reported in one study, as well as sweet foods in two studies. A decrease in soft drinks intake was reported in two studies [19]. These findings were in line with the results of this study. However, a detailed comparison with these studies is difficult due to different designs and outcome measures. Additionally, no comparable Portuguese data among similar age groups are available as far as we know [41,42]. 

Children’s attitudes changed positively immediately following the participative activity of each learning module. In all participatory activities, children’s motivation for healthy changes was observed, which was encouraging. It was also noteworthy that there was a great agreement between the behaviour changes observed in the current study and children’s attitudes expressed in participation of the activities. Preferences and expectations can be helpful information to both deepen knowledge about children’s trends and create opportunities for the continuous improvement for more effective intervention. For example, the low level in reported vegetable preferences can leave the impression that a wide variety of these foods was never tasted. Furthermore, the high level of willingness to try sports is encouraging, providing a clear sign that implementing various dynamics in the active living field into studies is well received by children. 

The learning modules and the participatory activities, in which children set goals and made commitments to change a specific behaviour or engage in new behaviour, resulted in significant eating changes. Finding out children’s needs, focusing on specific aspects of their behaviour that they were already prepared to change, and implementing practical recommendations appeared to be a successful strategy. Moreover, participatory methods provided children’s positive feedback; that they could change their daily behaviour and were responsible for their decisions.

In designing the intervention, three elements of the programme seemed essential to the achievement of significant results: behaviour commitment based on the TTM of stages change, the participatory activities included in the learning modules, and children involved in their decision-making goals and feedback. Children at these ages made numerous decisions about their diet; nevertheless, there was a general difficulty of taking the right decisions for everyday healthy eating even with adults. Therefore, the learning modules were designed to convert healthy eating principles into practical and specific behaviour, but regular guidance was required to support children for change behaviour. Furthermore, implementing the programme with this design allowed for several possibilities for improving the programme to further tailor it to the target population. 

This study has several strengths. It is one of the first Portuguese studies to evaluate the impact of a behavioural change programme over an entire school year. No Portuguese studies, to our knowledge, have reported any intervention approach based on TTM of stage change for the eating behaviour among children in early adolescence. The controlled design with baseline and post-intervention measurements, based on the repeated time–series design, allowed for the observations over the time period of one school year with the inclusion of eight different learning modules that would not have been possible using the single pre- and post-intervention design. Another strength of this study is that data analyses were based on a balanced sample by age and gender parameters. An additional strength was that the intervention was conducted in a real-world school setting and showed it could be included in the school organisation, implemented in different school subjects with different stakeholders.

Although the sample was ample, a larger sample may have resulted in more statistically significant results. Indeed, it was expected that all the available children could be included, but some did not deliver the baseline evaluation, (FR0) or some subsequent food records (inclusion criteria of this study), which led to the withdrawal of several tens of children records. 

Several factors may have influenced these results, as this study was not without limitations. First, randomisation was not possible, and the study was conducted under the constraints of school-based research without a concurrent control group submitted to the 3-day food records. Second, the study was restricted to only one school, and results may not have been generalised to other populations. Third, results were not free of selection and attrition bias, considering that children who participated in the study may have been the most motivated to change their behaviour. Finally, dietary intakes were self-reported and underreporting was a widespread problem in this research field. Indeed, measuring eating behaviour was relatively labour-intensive and not free of expectancy bias, although the 3-day food record was considered the “gold standard” for monitoring eating behaviour [31].

Despite the aforementioned limitations, the current published research on developing an effective and sustainable intervention to improve children’s eating behaviour in Portugal is scarce (e.g., [41,43,44]). It is expected that the present results can have important implications for improving the design of other school programmes that are intended to promote healthy eating and active living in young people. 

In addition, participatory activities allowed for the documentation of children’s attitudes and behaviour changes, which were of great value for this research. Concerning gender differences, this factor was not an important enough component in this analysis to draw strong conclusions about gender differences. Indeed, boys and girls had different food preferences and perceptions; therefore, aspects concerning gender differences should be the subject of careful research. 

This study supports preliminary evidence that PHS-pro may be effective in promoting behaviour changes to improve children’s eating patterns for preventing overweight and obese children. The failure to obtain some positive results in children’s vegetables consumption indicates that PHS-pro strategies need to be improved effectively in a future PHS-pro implementation. Nevertheless, this study raises several interesting ideas for future research to extend the programme to other ages and promote healthy eating over adolescence, coordinating an ongoing intervention tailored to real population needs. 

Finally, this pilot research did not involve changes in children’s family environment, particularly because of the time and conditions allocated to the research, but it is one important issue to be taken into account in further research. 

## 5. Conclusions

The learning modules and participatory activities designed for the PHS-pro implementation, in which children set goals and made commitments to change a specific behaviour or engage in a new behaviour, resulted in significant eating behaviour changes. Finding out children’s needs, focused on specific aspects of their behaviour that they were already prepared to change and the implementation of practical recommendations appeared to be a successful strategy. Thus, we conclude that applying these three elements: behaviour commitment based on the TTM of stages change, participatory activities, and children involved in decision-making goals and feedback, seems essential to achieve significant results and a promising intervention strategy.

## Figures and Tables

**Table 1:** Learning modules of the intervention and related educational and behaviour change goals.

Educational Components	Educational Goals	Behaviour Change Goals and Expected Outcomes in the Food Record
Learning module-one (LM1): “10 steps to be healthier” (i) Basic principles of healthy eating presentation in a simple 10 stepwise format (ii) In-group discussion to explore obstacles, solutions/options to meet the goal steps Activity-one (A1): Choosing the step, changing for better - Election of the most difficult step between the 10 wise steps and one step to improve as a behaviour change goal.	To reinforce healthy eating and active living; To recognize short and long-term benefits; To enhance motivation for changing behaviour; To identify main obstacles for a healthy eating; To encourage daily attitudes for positive changes.	Choosing a personal behaviour change goal Setting one practical measure to implement on daily behaviour Children’s commitment to accomplish the goal in the next days of the learning module Expected outcomes in Food Record 1 (FR1) - To find behaviour changes regarding the children’s selection steps: step 2 (starting lunch and dinner by eating vegetable soup), and step 7 (avoiding fried foods).
Learning module-two (LM2): Water & milk help you to grow up (i) Food items were identified (water, milk and yogurt) and their benefits for a healthy growth were described (ii) Discuss the pros and cons of beverages usually consumed: nutrients, prices, quantities and comparative nutritional contents Activity-two (A2): Favourite beverage of the month - Setting a solution to be achieved in the group	Calling for consumption of healthy choices: water, milk and yogurt, which contribute to a proper growth; Identification of benefits and adequate portions; Recommendations to decrease/moderate consumption of sugar-sweetened soft drinks;	Children resolution: adopting water or milk instead of soft drinks Expected outcomes in Food Record 2 (FR2): - Increasing water consumption and milk, and decreasing of sugar-sweetened soft drinks.
Learning module-three (LM3): Training everyday to be healthier (i) Introducing the concept of health: what it is to be healthy and its relationship with the benefits of health promotion and the influence of nutrition and food choices. (ii) Basic discussion around concepts of health Activity-three (A3): Planning for a healthier family - Designing a strategy for changes at home to a healthier family. A card was delivered to request each child to pay attention to their family’s lifestyle during the Christmas holidays.	Raising awareness of being healthy by giving priority to health choices and behaviour; Identifying unhealthy behaviour in the family’s lifestyle; Finding practical measures to implement a healthy environment at home.	Children were invited to design what would they consider to change their families’ behaviour to have a healthier life. No Food Record due to the Christmas season.
Learning module-four (LM4): 3 fruits a day, how much good it does? (i) Daily fruit intake benefits (ii) Recommendation intake: 3 portions of fruit per day (iii) Demonstration of ways of eating fruit (iv) Ranking the fruits’ top list Activity-four (A4): My Fruit planning: take a step forward into the future - Recording the fruit portion intakes from the day before, the present day and which fruits children prefer to eat on the day after or even if they would prefer to taste a new one.	Focusing on two key-points: the great variety of fruits available, and to respect each other’s preferences; Understanding differences and characteristics of different fruits; Identifying obstacles to fruit consumption, opportunities to increase the daily intake of fruit, and ways to add fruit to the diet; To raise awareness for daily fruit recommendations: compare the usual consumptions to increase children perception on their consumption.	Setting a specific daily fruit serving goal to be accomplished before the next module. Expected outcomes in Food Record 4 (FR4): Increasing fruit consumption.
Learning module-five (LM5): Fruits & vegetables are essential to life (i) The nutritional role of fruits and vegetables in the daily diet: benefits of nutrients, nutritional value, and richness in fibre. Activity-five (A5): The most popular ones: soup, salad and side dishes - Recording the favourite vegetables and ranking the most popular soup, salad and side dishes. The intention was to make children understand that they can have preferences for vegetables in the big vegetables family and can eat their favourite vegetables to reach a healthy goal.	Calling for the consumption of fruits and vegetables in the daily diet; Recognizing the existing plethora of vegetables (legumes, roots and tubers, pulses); Showing different ways of eating vegetables (soups, salads, and ways of preparation); Encouragment for choosing the favourite ones; Making children understand that they can have preferences for vegetables in the big vegetables family and can eat their favourite vegetables to reach a healthy goal.	Choosing a personal behaviour change goal. Setting one practical measure to implement on daily behaviour. Children’s commitment to accomplish the goal before the next learning module. Expected outcomes in Food Record 5 (FR5): Increasing intake of vegetable soup, vegetables as legumes or salads at meals, and increasing fruit consumption.
Learning module-six (LM6): Start on moving! (i) The importance of the regular practice of physical activity (PA) (ii) In-group discussion to explore obstacles which blocked the daily habit of practicing PA (iii) Explore solutions/options to try new sports Activity-six (A6): What sort of things do you play on? Recording the sports or playful activities usually practised by children and activities they would like to try.	Enhancing the health benefits of PA for self-image; Raising awareness to try new sports; Reinforcing physical activity classes of extracurricular programs at school.	Choosing a personal behaviour change goal to begin the practice of a sport or other forms of PA. No Food Record evaluation.
Learning module-seven (LM7): The best snacks (i) Food items that might be included as a healthy snack (ii) Demonstration of ways to change the quality of snacks, and how to confirm this using label readings (iii) In-group discussion to identify the most consumed foods during snack-breaks and arguments of what a snack must have to be healthy, that it must be rich in essential nutrients. Activity-seven (A7): Small meals big impact - Recording which is the best snack (card 1) and after the LM7, what a snack must have to be healthy (card 2). - Understanding the difference between which is considered the best snack and the best choice for a healthy snack.	Calling for the consumption of healthy foods during snack-breaks; Reinforcing skills that could help children become aware of their autonomy in food choices and eating behaviour (individual decision is exclusively the option of each child); Guiding how to choose a healthy snack in various aspects: nutrients, preferences, prices and new options; Encouraging one specific eating habit to change during breaks and to improve nutritional snacks: breaks are an opportunity to add fruit.	Choosing a personal behaviour change goal for healthy alternatives to snack breaks. Implementing a snack rich in essential nutrients. Children’s commitment to accomplish the goal before the next module. Expected outcomes in Food Record 7 (FR7): Evolution in the nutritional quality of food choices of snacks: increasing fruit, bread, yogurt and milk consumptions, and decreasing soft drinks, high-energy dense food (croissants, panikes, donuts, cakes, pastries, cookies and biscuits).
Learning module-eight (LM8): The final game: who has learned about everything? (i) Closure of the intervention (ii) Children were invited to play a little game. This activity allowed children’s self-evaluation towards their behaviour changes to their eating habits over the programme. Activity-eight (A8): The final game: who has learned about everything? Game—‘let’s play on: who has learned about everything?’	Recalling and re-examining concepts and self-assessment regarding changes over the intervention; Reinforcing skills for healthy eating and active living; To enhance motivation for changing behaviour; To encourage daily attitudes towards positive changes.	No application of a food record. Application of a small questionnaire to ask children whether they could identify any changes that occurred in their eating and lifestyle habits over the programme.

**Table 2:** Baseline eating behaviour of children in a 3-day period (*n* = 157).

Reported Servings	0	1	2	3	4	5 or More
	% of Children					
Fruit	31.2	17.2	17.8	12.7	7.0	14.7
Vegetable soup	36.9	26.8	12.7	10.8	5.1	7.7
Vegetables (in salad or cooked)	51.0	28.0	10.8	6.4	2.5	1.3
Milk products	1.9	1.3	6.4	13.4	15.9	61.0
Bread	4.5	12.7	14.0	24.8	19.7	24.2
High-energy dense food	17.8	25.5	27.4	19.1	4.5	5.5
Fried food	38.2	39.5	14.6	5.7	1.9	0.0
Water	14.6	21.0	18.5	14.6	12.7	18.4
Soft drinks	49.0	22.3	11.5	7.0	3.8	6.2

**Table 3:** Comparison of outcomes between baseline and post-intervention 3-day food records (FR1, FR2, FR4, FR5 and FR7).

Food behaviour evaluation between baseline and FR1				
Outcome	Sample size	Baseline	3-day FR1	
Consumption (servings)		Med (IQR)	Med (IQR)	*p*-value
Vegetable Soup	*n* = 143	1 (0–2)	2 (0–3)	0.003 *
Fried food		1 (0–1)	1 (0–2)	0.673
Food behaviour evaluation between baseline and FR2				
Outcome	Sample size	Baseline	3-day FR2	
Consumption (servings)		Med (IQR)	Med (IQR)	*p*-value
Milk products	*n* = 145	5 (4–7)	5 (4–7)	0.024 *
Water		2 (1–4)	2 (0–4)	0.435
Soft drinks		1 (0–2)	0 (0–2)	0.340
Food behaviour evaluation between baseline and FR4				
Outcome	Sample size	Baseline	3-day FR4	
Consumption (servings)		Med (IQR)	Med (IQR)	*p*-value
Fruit	*n* = 138	2 (0–3)	2 (0–4)	0.008 *
Food behaviour evaluation between baseline and FR5				
Outcome	Sample size	Baseline	3-day FR5	
Consumption (servings)		Med (IQR)	Med (IQR)	*p*-value
Vegetables (in salad or cooked)	*n* = 129	0 (0–1)	0 (0–1)	0.400
Vegetable Soup		1 (0–3)	1 (0–3)	0.093
Fruit		2 (0–3)	2 (0–4)	0.903
Food behaviour evaluation between baseline and FR7				
Outcome	Sample size	Baseline	3-day FR7	
Consumption (servings)		Med (IQR)	Med (IQR)	*p*-value
Bread	*n* = 127	3 (2–5)	3 (2–4)	0.455
High-energy dense food		2 (1–3)	1 (0–2)	0.048 *
Milk products		5 (4–7)	5 (3–6)	0.078
Soft drinks		1 (0–2)	0 (0–1)	0.042 *
Fruit		2 (0–3)	1 (0–3)	0.176
Med (IQR): median (interquartile range).				
Wilcoxon Rank Test *: *p* < 0.050				

**Table 4:** Fruit servings consumed in two moments (previous day and assessment day), and fruit eating intentions (A4).

Fruit Servings	0	1	2	3	4
	% of Children				
Previous day	7.7	23.9	35.9	23.9	5.6
Assessment day	67.6	25.4	6.3	0.7	0
Next day intentions	0	10.6	24.6	49.3	11.3

**Table 5:** Vegetable preferences on the basis of vegetable soup, salad and vegetable side dishes (A5).

Number of Preferences	0	1	2	3	4
	% of Children				
Vegetable soups	0	17.3	78.2	3.2	0.6
Fresh salads	3.2	23.1	66.7	5.1	1.9
Vegetable side dishes	1.9	14.1	68.6	8.3	3.8

**Table 6:** Frequency distribution of children practising sports or playful activities (A6).

Practice Daily Physical Activities	
	% of Children
Nothing	56.6
Sports	43.4
Physically active at school (playground)	
Playing games	51.3
Walking	25.0
Football	21.1
Dancing	2.6
Physically active after school	
Cycling	23.7
Football	21.1
Walking	14.5
Other sports	13.2
Playing games	11.8
Nothing	11.8
Dancing	3.9
Physically active at weekend (in the family)	
Going for a walk	44.1
Cycling	28.9
Nothing	25.7
Swimming	1.3
Expectations of trying sports or playful activities	
Sports	65.8
Playful activities	31.6
Nothing	2.6

**Table 7:** Frequency distribution of children’s healthy food choices for snacks (A7).

Food Choices for Snacks	A7-Card-One	A7-Card-Two
	% of Children	
Healthy foods	81.9	95.5
Unhealthy foods	18.1	4.5

## Data Availability

The data presented in this study are available on request from the corresponding author. The data are not publicly available due to data privacy of the participants.
